# Seasonal food habits and prey selection of Amur tigers and Amur leopards in Northeast China

**DOI:** 10.1038/s41598-018-25275-1

**Published:** 2018-05-02

**Authors:** Haitao Yang, Hailong Dou, Raj Kumar Baniya, Siyu Han, Yu Guan, Bing Xie, Guojing Zhao, Tianming Wang, Pu Mou, Limin Feng, Jianping Ge

**Affiliations:** 1 Ministry of Education Key Laboratory for Biodiversity Science and Engineering, State Key Laboratory of Earth Surface and Resource Ecology, College of Life sciences, Beijing Normal University, Beijing, 100875 China; 2 College of life sciences, Qufu Normal University, Qufu, 273165 China

## Abstract

We analyzed the scats of Amur tigers and Amur leopards, and examined their annual and seasonal food habits in Northeast China to comprehend their coexistence. Wild boar had the highest annual and seasonal consumption frequencies by the tigers, while both roe deer and sika deer were mostly preyed by the leopards annually. The three species appeared to be the key preys in terms of high proportion of consumed biomass by the two felids. Our data also revealed numerous mid-sized carnivores and small mammals included in the two felids’ food list. We used the relative abundance and biomass density estimation in prey density estimation to calculate the prey preferences of tigers and leopards, and both methods confirmed that Amur tigers strongly preferred wild boar. However, preference estimations of Amur leopards were not consistant, or even opposite to one another from the two methods. The results of the study suggested that prey preference of predators is largely determined by body size of the prey species. Variation in diet composition of the two felids suggests that resource partitioning may contribute to their coexistence.

## Introduction

Both Amur tiger (*Panthera tigris altaica*) and Amur leopard (*Panthera pardus orientalis*) are endangered felids on the list of IUCN and the subspecies of both genera distributing in the northernmost of the ranges of the species. Habitat loss, landscape fragmentation, prey depletion and poaching have contributed to the decline of the Amur tiger from more than 3,000 to about 500 during the past century^1–3^, while the Amur leopard, to less than 100 individuals^4^. The Amur tigers are currently existing in two separate populations: a larger one in the Sikhote-Alin Mountains of Russia (415–490 individuals)^5,6^, and a much smaller one, almost isolated from the larger one, in southwestern Primorsky Krai, Russia, and the nearby Hunchun region in China^4,7,8^. The Amur leopards occur as a small single population co-existing with the smaller tiger population^6,9,10^.

Prey availability is one of the principal drivers of tiger and leopard distributions and abundance^11^. Tigers and leopards prefer prey species of their own sizes^12,13^, suggesting that preferred species of the Amur tiger and Amur leopard are likely different, thereby reducing competition for resources and increasing the likelihood of co-existence^14–17^. Investigation of their food habits and prey selections would provide a better understanding of the predator-prey relationship and help define conservation priorities for both felids.

Food habits and prey selection of Amur tigers and leopards have been widely studied^3,18,19^, mostly based on kill sites found by snow tracking or telemetry. Due to the difficulties of finding kill sites of small animals, such methods would overestimate large prey items in the diet^14,20^. A widely used field method for examining predator diets is to identify undigested parts (hair, hoof or tooth) from scats of the predators comparing with the reference of potential food items^15,20–24^. This is a more objective approach with less bias. Furthermore, faecal analysis method is relatively easier in obtaining samples with non-invasive procedure^15^.

In Northeast China and Far East Russia, available prey in terms of secondary productivity is lower than in more productive habitats in the tropical and subtropical areas, a fact that may aggravate competition between the two native carnivores. Both are “ambush” predators (versus cursorial predators)^25^ that increase the likelihood of dietary overlap, despite the differences in body sizes. In Russian Fareast, it has been reported that Amur leopard had more diverse prey than Amur tiger did^20,24^. While such studies are rare in Northeast China.

In this study, we used scats collected from the tiger/leopard areas of Northeast China to explore their prey profiles for the following objectives: (1) to determine the seasonal frequency of occurrence and relative biomass contribution of the principal prey species in predator diets; (2) to evaluate the seasonal difference in prey selection using different methods in estimating prey availability-the relative abundance and biomass density methods; (3) to examine the diet profiles to comprehend resource partitioning and ecological sympatry of the tigers and the leopards in the study area of Northeast China. We hypothesized that the resource utilization of tigers and leopards would be partitioning in their sympatric range in order to reduce the competition.

## Results

After excluding the scats composed of grass, sands, and unidentifiable objects, we had a total of 217 valid tiger scats (80 in summer, 115 in winter and 22 no detailed time records), and 52 leopard scats (23 in summer, 25 in winter and 4 no detailed time records) collected from January 2014 to December 2016. Among them, all contain one prey item, but 29 tiger scats and 7 leopard scats that contained two prey items.

Camera trap data from August 2014 to January 2015 were used to calculate the relative abundance index (RAI)^26^ of three main prey species as the relative availability of the prey. The RAI values for roe deer (*Capreolus pygargus*) were the highest in both winter (1.947) and summer (4.459), while the RAIs for sika deer (*Cervus nippon*) (0.548 and 1.835 in the winter and summer, respectively) and wild boar (*Sus scrofa*) (0.757 and 1.539 in winter and summer, respectively) were lower (Table 1).

**Table 1:** The annual and seasonal relative abundance index (RAI and biomass) and biomass densities proportion for wild boar, sika deer and roe deer in NE China.

Species		Density of prey species					
		Annual		Winter		Summer	
		RAI	Abundance proportion (%)	RAI	Abundance proportion (%)	RAI	Abundance proportion (%)
Wild boar		1.081	21.10	0.757	23.29	1.539	19.64
Sika deer		1.069	20.86	0.548	16.84	1.835	23.43
Roe deer		2.975	58.04	1.947	59.87	4.459	56.93
	**Weight (kg)**	**Biomass (kg/km** ^**2**^ **)**	**Abundance proportion (%)**	**Biomass (kg/km** ^**2**^ **)**	**Abundance proportion (%)**	**Biomass (kg/km** ^**2**^ **)**	**Abundance proportion (%)**
Wild boar	103	125.076	31.64	74.695	29.72	176.531	32.65
Sika deer	95	168.153	42.54	113.165	45.02	223.334	41.31
Roe deer	37	102.038	25.82	63.50	25.26	140.769	26.04

Note: RAI is the relative abundance index from the camera trap data; biomass was also calculated from the camera trap data based on Random Encounter Model.

The biomass of the three ungulate species calculated based on Random Encounter Model^27^ indicated that sika deer had the highest biomass densities (113.165 kg/km^2^ and 223.334 kg/km^2^ in the winter and summer, respectively) and the abundance proportions (45.02% and 41.31% in winter and summer, respectively). These were different from that calculated from the RAI values, that roe deer had the highest abundance proportions (59.87% and 56.93% in winter and summer, respectively) (Table 1). Based on biomass density, the seasonal abundance proportions of wild boar and sika deer were actually higher (Table 1).

### Food habits

We identified 11 prey species in the tiger scats, and 11 from the leopard scats (Table 2) in annually total. The prey compositions were significantly different (χ^2^ = 38.524, *p* < 0.01, Fisher’s exact test) between the tigers and the leopards. Wild boar, roe deer and sika deer were all the most common prey species for the tigers (73.97%) and the leopards (75.00%), and the three species significantly differed (χ^2^ = 26.254, *p* < 0.01, Fisher’s exact test) in the diets of the tigers and the leopards. Wild boar was most common in the tiger scats (36.64%). Roe deer was most common in the leopard scats (37.50%); but the second commonly preyed by the tigers (22.12%). Sika deer was the second most common prey item of the leopards (26.92%) (Table 2). Mid-size carnivores and small mammals were also consumed by both big cats (Table 2). The three ungulate species comprised 80.22% and 76.65% of overall biomass intake of the tigers and the leopards, respectively (Table 3). Wild boar contributed the greatest biomass for the tigers (45.88%) while sika deer, the largest consumed by leopards (33.71%) (Table 3).

**Table 2:** Number of prey items and proportion (%) of different prey species in the annual and seasonal diets of Amur tiger and Amur leopard in NE China.

	Amur tiger		Amur leopard	
	n.	proportion occurrence (%)	n.	proportion occurrence (%)
**Annual**				
Wild boar	79.5	36.64	5.5	10.58
Roe deer	48	22.12	19.5	37.50
Sika deer	33	15.21	14	26.92
Badger	9.5	4.38	3.5	6.73
Black bear	6	2.76	—	—
Raccoon dog	18	8.29	1.5	2.88
Red fox	7.5	3.46	2	3.85
Hare	6	2.76	1.5	2.88
Dog	5.5	2.53	0.5	0.96
European otter	—	—	1	1.92
Musk deer	1	0.46	1	1.92
Cattle	3	1.38	2	3.85
**Summer**				
Wild boar	19.5	24.38	4.5	19.57
Roe deer	25	31.25	7	30.43
Sika deer	13.5	16.88	3.5	15.22
Badger	3	3.75	2	8.70
Black bear	2	2.50	—	—
Raccoon dog	6.5	8.13	1.5	6.52
Red fox	3.5	4.38	0.5	2.17
Hare	5	6.25	0.5	2.17
Dog	2	2.50	0.5	2.17
European otter	—	—	1	4.34
Musk deer	—	—	1	4.34
Cattle	—	—	1	4.34
**Winter**				
Wild boar	52.5	45.65	1	4.00
Roe deer	20.5	17.83	11.5	46.00
Sika deer	13	11.30	7.5	30.00
Badger	4	3.48	1.5	6.00
Black bear	3	2.61	—	—
Raccoon dog	11	9.57	—	—
Red fox	4	3.48	1.5	6.00
Hare	0.5	0.43	1	4.00
Dog	2.5	2.17	—	—
Musk deer	1	0.87	—	—
Cattle	3	2.61	1	4.00

**Table 3:** Proportion biomass contribution (D) of prey species to Amur tiger and Amur leopard annual and seasonal diets in NE China.

	weight(kg)	Y (kg/scat)	Amur tiger	Amur leopard
			D (SE)	D (SE)
**Annual**				
Wild boar	103	5.59	45.88 (3.60)	13.94 (5.42)
Roe deer	37	3.28	16.25 (2.17)	29.00 (6.11)
Sika deer	95	5.31	18.09 (2.66)	33.71 (7.20)
Badger	6	2.19	2.15 (0.65)	3.48 (1.68)
Black bear	150	7.23	4.48 (1.78)	—
Raccoon dog	5	2.16	4.01 (0.89)	1.47 (1.10)
Red fox	5.4	2.17	2.16 (0.86)	1.97 (1.24)
Hare	2	2.05	1.68 (0.59)	1.39 (1.05)
Dog	31	3.07	1.74 (0.72)	0.70 (1.02)
European otter	6	2.19	—	0.99 (1.02)
Musk deer	10	2.33	0.24 (0.24)	1.06 (1.03)
Cattle	331	13.57	4.20 (2.33)	12.31 (7.61)
**Summer**				
Wild boar	103	5.59	33.88 (5.87)	26.19 (10.33)
Roe deer	37	3.28	25.49 (4.56)	23.90 (8.63)
Sika deer	95	5.31	22.28 (5.09)	19.35 (8.95)
Badger	6	2.19	2.04 (1.09)	4.56 (2.93)
Black bear	150	7.23	4.49 (3.08)	—
Raccoon dog	5	2.16	4.36 (1.62)	3.37 (2.60)
Red fox	5.4	2.17	2.36 (1.21)	1.13 (1.17)
Hare	2	2.05	3.19 (1.35)	1.07 (1.12)
Dog	31	3.07	1.91 (1.35)	1.60 (1.66)
European otter	6	2.19	—	2.28 (2.40)
Musk deer	10	2.33	—	2.43 (2.53)
Cattle	331	13.57	—	14.13 (11.66)
**Winter**				
Wild boar	103	5.59	53.91 (4.97)	5.31 (5.12)
Roe deer	37	3.28	12.35 (2.56)	35.82 (9.89)
Sika deer	95	5.31	12.68 (3.01)	37.82 (10.60)
Badger	6	2.19	1.61 (0.76)	3.12 (2.39)
Black bear	150	7.23	3.98 (2.24)	—
Raccoon dog	5	2.16	4.36 (1.26)	—
Red fox	5.4	2.17	1.59 (0.76)	3.09 (0.08)
Hare	2	2.05	0.19 (0.19)	1.95 (0.06)
Dog	31	3.07	1.41 (0.85)	—
Musk deer	10	2.33	0.43 (0.43)	—
Cattle	331	13.57	7.48 (3.96)	12.89 (10.91)

Note: Y is the correction factor; The standard error (SE) of each prey item were generated using 10000 bootstrap simulations.

Nine prey items were identified in the tiger diet and 11 items in the leopard diet in the summer (Table 2). In the winter, 11 and 7 items in the tiger and leopard diets, respectively (Table 2). Although Fisher’s exact test indicated a significant difference between the tiger and leopard diets in both winter (χ^2^ = 74.645, *p* < 0.01) and summer (χ^2^ = 27.474, *p* < 0.01), wild boar, roe deer and sika deer in combine provided the majority of biomass consumption in the summer (81.65% and 69.44% for the tigers and the leopards, respectively) and the winter (78.94% and 78.95% for the tigers and the leopards, respectively) (Tables 2 and 3). Nonetheless, the overall proportions of the prey species in the biomass consumptions by the tigers and the leopards differed significantly in the two seasons (χ^2^ = 14.586, *p* < 0.05 for the tigers and χ^2^ = 25.136, *p* < 0.01 for the leopards).

### Prey selection

The annual prey selection by Amur tiger showed a significant prey preference (*p* < 0.05) for wild boar based upon both RAI (Jacobs’ index = 0.562) and biomass (Jacobs’ index = 0.347) results (Table 4). The results from RAI and biomass, however, differed for sika deer and roe deer. Based on biomass, the tigers had a significant avoidance (*p* < 0.05) of sika deer, but no significance found according to RAI. For roe deer, RAI results showed the tigers had significant avoidance (Jacobs’ index = −0.537, *p* < 0.05), whereas biomass results indicated otherwise (Jacobs’ index = 0.090). Prey selections by the leopard also differed between RAI and biomass results. A significant avoidance (*p* < 0.05) for wild boar was revealed by the biomass result, but not shown such by the RAI results. Nonetheless, RAI results showed a preference of sika deer and avoidance of roe deer, but the biomass results showed an avoidance trend of sika deer, but a significant preference for roe deer (*p* < 0.05) (Table 4).

**Table 4:** Jacobs’ index scores and associated 95% confidence intervals (CI) based on relative abundance index and biomass measuring tiger and leopard annual and seasonal preferences and avoidances for three main prey species in NE China.

			Amur tiger			Amur leopard		
			Wild boar	Sika deer	Roe deer	Wild boar	Sika deer	Roe deer
Annual	RAI	Jacobs’ index	**0.562**	0.027	**-0.537**	-0.266	0.373	-0.161
		95% CI	**0.447 – 0.651**	-0.165 – 0.191	**-0.646 – -0.419**	-0.678 – 0.126	-0.042 – 0.567	-0.455 – 0.112
	Biomass	Jacobs’ index	**0.347**	**-0.454**	0.090	**-0.498**	-0.124	**0.484**
		95% CI	**0.185 – 0.455**	**-0.601 – 0.313**	-0.083 – 0.245	**-0.801 – -0.147**	-0.449 – 0.125	**0.157 – 0.651**
Summer	RAI	Jacobs’ index	**0.354**	0.021	**-0.293**	0.231	-0.050	-0.199
		95% CI	**0.065 – 0.542**	-0.348 – 0.252	**-0.513 – -0.059**	-0.405 – 0.607	-0.677 – 0.547	-0.650 – 0.249
	Biomass	Jacobs’ index	**0.326**	**-0.433**	0.084	-0.107	-0.357	0.430
		95% CI	**0.161 – 0.442**	**-0.562 – -0.290**	-0.084 – 0.245	-0.648 – 0.347	-0.827 – 0.050	-0.114 – 0.724
Winter	RAI	Jacobs’ index	**0.662**	-0.013	**-0.657**	**-0.717**	0.505	-0.056
		95% CI	**0.526 – 0.762**	-0.197 – 0.223	**-0.783 – -0.513**	**-1.000 – -0.291**	-0.097 – 0.736	-0.577 – 0.253
	Biomass	Jacobs’ index	**0.559**	**-0.612**	-0.044	**-0.789**	-0.142	**0.596**
		95% CI	**0.387 – 0.690**	**-0.764 – -0.440**	-0.314 – 0.149	**-1.000 – -0.435**	-0.662 – 0.239	**0.193 – 0.762**

Note: Tiger and leopard diets were estimated from scats, and relative abundance and biomass were calculated from camera trap data. Confidence interval of each prey item was generated using 10000 bootstrap simulations. 95% CI that do not overlap with zero indicate significant (*p* < 0.05) preference or avoidance for a prey species.

Seasonal variation in prey selection was also reflected in the results. Both RAI and biomass based results showed the tigers preferred wild boar in the winter and summer (*p* < 0.05) (Table 4). The RAI results showed that the tigers avoided roe deer in both seasons (*p* < 0.05), and avoided sika deer in the winter but preferred it in the summer (Table 4). The biomass results revealed that Amur tiger significantly avoided sika deer in both seasons; preferred roe deer in the summer, but avoided it in the winter (Table 4). In the summer, the Amur leopard did not show a significant preference or avoidance to the prey species (Table 4). In the winter, it preferred sika deer but avoided wild boar and roe deer according to the RAI results that differed somewhat from biomass based results.

## Discussion

### Seasonal diets of Amur tigers and Amur leopards

Our dietary analysis revealed wild boar, sika deer and roe deer as the primary prey consumed by the tigers and the leopards with some seasonal variations. Our results were along with that found in Russian Far East^20,24^.

Wild boar and roe deer were the most common item consumed by tigers and leopards, respectively (Table 2). Wild boar was the principal prey species for tigers, with lower consumption in the summer comparing to that in the winter (Tables 2 and 3). The leopard diet had an opposite trend with higher occurrence frequency and biomass intake of wild boar in the summer than in the winter (Tables 2 and 3). We hypothesize that leopards avoid adult wild boar and prefer smaller individuals more available after spring birthing^14,15^. We speculate that the tigers may prefer to adult prey to maximize their energetic return^15,17^.

The frequency of occurrence and relative biomass contributions of roe deer and sika deer were varied in the tiger and leopard diets; the biomass contribution of sika deer in the leopard winter diet was the highest in China, similar to those found in Russia^24^, but the proportion of sika deer in China was lower (Table 3), probably due to the bizarre distribution of sika deer concentrated along the Sino-Russia^6,28^, Amur leopards in China relied more on roe deer than in Russia. The proportion of the smaller animals in the leopard winter diet was lower than in its summer diet, probably due to hibernation of both badger (*Meles leucurus*) and raccoon dog (*Nyctereutes procyonoides*) that may strengthen the predation on sika deer and roe deer during the winter. The prey diversity of the leopard was generally higher than that of the tiger^20,24,29^, but our results showed otherwise probably due to the small sample size of leopard scats.

Domestic animals had the smallest biomass proportion among the prey consumed by the Amur tigers and the Amur leopards. Our results showed that they consumed relatively more domestic animals in China than in Russian^20,24^ probably due to higher availability. Despite the small proportions, domestic animals are not ignorant component in the tiger and leopard diets in China, especially for the tigers in the winter. Due to the increased difficulty in tracking and hunting secluded prey in the winter, the tigers may inevitably turned to available domestic animals such as cattle^28^.

### Prey selection

Accurate estimation of prey density is of great importance^30^ in studying prey selection. In this study, RAI^26^ and biomass from the Random Encounter Model^27^ were utilized to estimate the densities of the three main prey species. The two methods showed different results in the density of roe deer (Table 1) that could affect the assessment of prey selection (Table 4). Our results showed that wild boar was the most preferred prey of the tigers, in accordance with Russia studies^3,20,24,29^. Both RAI and biomass based results directed that the tigers avoided sika deer and roe deer (Table 4). The preference of wild boar by the Amur tigers could be attributed to its large body weight^12^, shorter status and poorer mobility, especially when the ground is covered with snow in the winter; moreover, compared to deer, predation on wild boar confers higher energy profits to tiger. Considering that a tiger population cannot be maintained in the absence of the most abundant ungulate species, the avoidance of sika deer by tigers is puzzling^12^. The biomass result showed that the Amur leopard depended heavily on roe deer and avoided wild boar (Table 4). Our RAI results for prey selection by tigers and leopards correspond to those facts obtained in Russia^3,20,24,29^. However, the biomass result showed a higher use of roe deer by the leopards, though the biomass proportion of sika deer was relatively higher in the winter. A previous study suggested that the leopards primarily feed on small to medium-sized prey (10–40 kg)^13^. Due to its smaller size, selection on roe deer over sika deer is an appropriate strategy for the leopards (Tables 1 and 4). In addition, due to the along-border distribution of sika deer, a portion of leopard scats collected in the region where sika deer is absent, that may bias the of prey selection estimation of the leopards^6,31^.

In this study, the body weight of prey species was an important factor in estimating the food habits and prey selections of the predators. Average body weight was used to estimate the biomass contribution of prey in the diet of the Amur tiger and the leopard^20,24^, which may result in a higher estimation of wild boar, because of the difficulties in identification of the sex and age of the prey species from scat analysis. Based on kill data, Miller, *et al*.^32^ reported that Amur tiger preferred adult red deer (*Cervus elaphus*), sub-adult and juvenile wild boars. The prey density of prey species calculation through relative abundance would influence the estimation of prey selection by the tigers and the leopards. The estimation of prey density is affected by the speed, group size and age structure of the prey species. Therefore, we employed a Random Encounter Model, which considered moving speed and group size as parameters in the estimation. The accurate measures of average speed, however, is still a challenge. We used the daily average moving distance instead, which may result in some bias in density estimation.

Our results strongly suggests that the body size of the prey species is an important factor in prey preference analysis for the tigers and the leopards. The diets differences between the felids indicate that resource partition enhance their coexistence.

## Materials and Methods

### Ethics statement

The State Forestry Administration of China has approved this study as a part of the long-term Tiger Leopard Observation Network (TLON). Jilin Provincial Bureau of Forestry and the Forestry Industry Bureau of Heilongjiang Province permitted the work. Beijing Normal University conducted this study in collaboration with the local administrations. Scats survey was carried out using non-invasive technology without direct contact with animals. Reference hair samples of prey species were obtained from the zoos or the confiscated illegal wildlife products. Our treatment of faecal samples followed Bassi, *et al*.^33^.

### Study area

**Figure 1:**
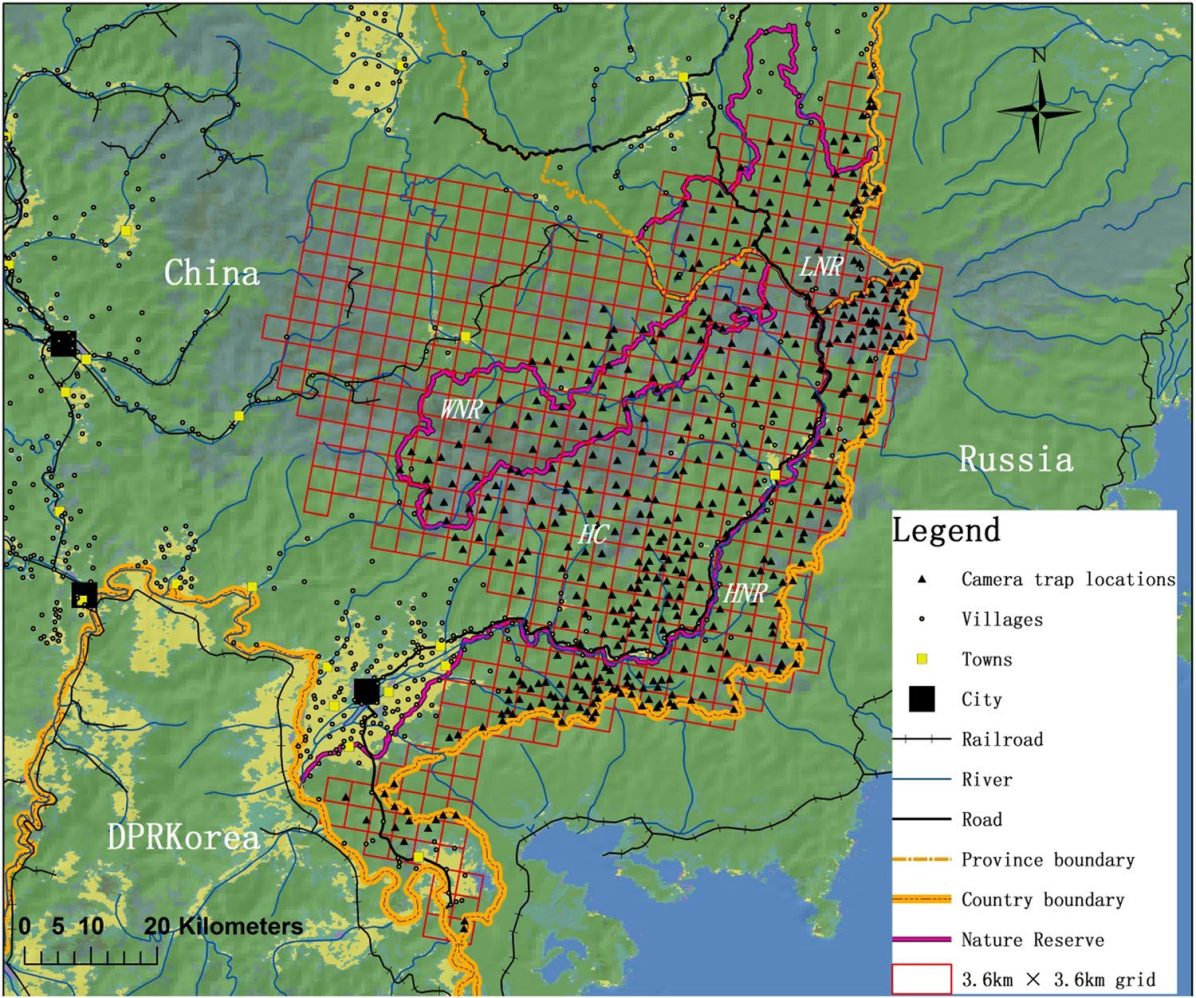
Study area and locations of camera trapping sites in Northeast China. Map was created using ArcGIS software by Esri (Environmental Systems Resource Institute, ArcGIS 10.1; www.esri.com).

We conducted our study in the eastern section of Jilin Province and the adjacent southeastern Heilongjiang Province, Northeast China^6,9^ (Fig. 1), bordering the southwest Primorsky Krai, Russia to the east and DPRK to the southwest. This region includes three nature reserves of China: Hunchun Nature Reserve (HNR), Wangqing Nature Reserve (WNR), Laoyeling Nature Reserve (LNR), and a large tract of unprotected forest and agricultural lands connecting the reserves (HC). The elevation of this area ranges 5 to 1,477 m^9^. The area was gridded in to grids of 3.6 × 3.6 km, and 483 camera traps in total were set in the grids (1 to 4 camera trap locations per grid) (Ltl Acorn 6210M, Shenzhen, China) onto the trees approximately 0.4–0.8 m above the ground. Camera traps were set to be active continuously, with a 1 minute delay between consecutive videos. The cameras were checked monthly to download videos and replace batteries. Consecutive videos of the same species within 0.5 h were not included in the data analysis to avoid inflated counts^26^.

The major sympatric carnivores or omnivores in this area are Amur tiger, Amur leopard, Asiatic black bear (*Ursus thibetanus*), brown bear (*Ursus arctos*) and Eurasian lynx (*Lynx lynx*). The potential prey species for the tigers and the leopards include wild boar, sika deer, roe deer, musk deer (*Moschus moschiferus*), red fox (*Vulpes vulpes*), badger, raccoon dog, Siberian weasel (*Mustela sibirica*), sable (*Martes zibellina*), yellow-throated marten (*Martes flavigula*), leopard cat (*Prionailurus bengalensis euptihurua*), hedgehog (*Erinaceus amurensis*), hare (*Lepus mandshuricus*), European otter (*Lutra lutra*) and domestic animal species (cattle, dog, goat, horse and cat).

### Field methods

From January 2014 to December 2016, scats of tigers and leopards were collected by field staff along the vehicular and logging roads, and ridges where the felids commonly deposit scats^14,20,34^. Identification of tiger and leopard scats was based on size, morphology, signs such as ground tracks or scrapes in the wild^14,20^. The scats were placed into plastic bags, sealed in the field and then stored refrigerated at −20 °C until analysis. Then species identification of tiger and leopard were successfully following by molecular methods^35,36^. The samples that failed in DNA amplification were excluded.

### Scat analysis

Due to extreme differences in weather and availability of prey in our study area, we were partitioned observations into two seasons: summer (from May to October) and winter when snow often covered the ground (from November to April). The two seasons coincided with availability of some prey species (e.g., species such as badgers, raccoon dogs, and chipmunks are true or partial hibernators in winter). Thus, food habits and prey selections were analysed annually and seasonally.

The tiger and leopard scats were first washed under running tape water until the undigested remains (hair, bone, teeth and hooves) were separated from other faecal materials. The remains were placed in the envelopes and oven-dried at 65 °C for 48 h^33^. Hair features (colour, length, thickness and medullary configuration) constitute the most important information for identifying prey consumed^14,34,37^. We chose 10 hairs from the remains to examine under a microscope by comparing with references obtained from the study area and a standard prey hair manual^14,15,20,22^.

We scored the scats as one for a single prey species identified. If two or more prey species were identified in a scat, it would be scored as a proportion of one (e.g. 0.5 each if two species were present). We used the correction factor derived by Ackerman, *et al*.^38^ to estimate biomass contribution to diets based on the scores in the scats as following (1):

1 $${\rm{Y}}=1.98+0.035{\rm{X}};$$

where Y (kg) is the weight of the prey consumed per scat and X (kg) is the average body weight of the prey species obtained from the literature^39–41^. The proportion of biomass contribution was estimated as following^42^ (2):

2 $${\rm{D}}=\frac{{Y}_{i}\times {A}_{i}}{\Sigma ({Y}_{i}\times {A}_{i})};$$

where D is the proportion of biomass contribution of prey species, *Y*_*i*_ is the correction factor for species *i*, and *A*_*i*_ is the frequency of occurrence of *i*^*th*^ item.

We only estimated prey preference for the three species most commonly found in scats (wild boar, sika deer, roe deer) in this study due to insufficient sample in smaller prey species. Jacobs’ index^30^ was utilized to assess the prey selection of Amur tiger and leopard valuing from −1 (strongly avoided) to +1 (strongly preferred). The density of a prey species is the most important in the estimation of Jacobs’ index, therefore, we used two methods to estimate the density: (1) RAI - the relative abundance index (the number of detections per 100 camera-trap days of every species)^20,24^; (2) the Random Encounter Model, it estimates animal density using camera traps data without individual recognition. We then calculated the available biomass of the three ungulates based on the absolute density estimation from the Random Encounter Model (biomass = density from Random Encounter Model * group size * average body weight of a prey species). The proportions of available ungulate biomass and RAI were used to calculate the prey selection of the tigers and the leopards. The Random Encounter Model assumes modelled species being a closed population, three months per period (August to October 2014 for summer and November 2014 to January 2015 for winter) were selected for the model to estimate the seasonal density of animals (3):

3 $${D}_{i}=\frac{{y}_{i}}{{\rm{t}}}\frac{\pi }{{v}_{i}r(2+\theta )};$$

where *D* is the density of prey species *i*; *y*_*i*_ is the detections of *i* species; *v*_*i*_ is the moving speed of *i* species, and we used 5.9 km/day^43^, 2.78 km/day^44^, and 3.8 km/day^45^ for wild boar, sika deer and roe deer, respectively; t is the total workdays of camera traps (Table S1); *r* (=0.005 km) is the detection radius of the camera traps (Ltl 6210, Shengzhen, China); and *θ* (=23.5°) is detection angle of the camera trap. The Jacobs’ index was calculated as (4):

4 $${\rm{J}}=\frac{({A}_{i}-{P}_{i})}{({A}_{i}+{P}_{i}-2{A}_{i}{P}_{i})};$$

where J is Jacobs’ index; *A*_*i*_ is the frequency of occurrence of *i* item; and the *P*_*i*_ is the abundance proportion of prey items *i* obtained by the camera survey (RAI) and the biomass proportion of prey items *i* based on the Random Encounter Model.

Fisher’s exact test was used to compare annual and seasonal differences in prey composition between Amur tiger and Amur leopard, and then to compare the seasonal differences for each felid species. The standard error (SE) and 95% CI of each estimation item of biomass contribution and prey selection index were generated using 10,000 bootstrap simulations.

## Electronic supplementary material

Table S1
