# Supplementary material for: Seasonal food habits and prey selection of Amur tigers and Amur leopards in Northeast China
Source: Sci Rep. 2018 May 2;8:6930. doi: 10.1038/s41598-018-25275-1 (PMC5931987; doi:10.1038/s41598-018-25275-1)
Supplement: Supplementary file 1 — Table S1 [file 41598_2018_25275_MOESM1_ESM.docx]

**Seasonal food habits and prey selection of Amur tigers and Amur leopards in Northeast China**

Haitao Yang ^a^, Hailong Dou ^a,b^, Raj Kumar Baniya ^a^, Siyu Han ^a^, Yu Guan ^a^, Bing Xie ^a^, Guojing Zhao ^a^, Tianming Wang ^a^ , Pu Mou ^a^, Limin Feng ^a,*^, Jianping Ge ^a^

^a^ *Ministry of Education Key Laboratory for Biodiversity Science and Engineering, State Key Laboratory of Earth Surface and Resource Ecology, College of Life sciences, Beijing Normal University, Beijing, 100875, China*

^b^*College of life sciences, Qufu Normal University, Qufu, 273165, China*

*Correspondent: Limin Feng

Address: School of life Science, No. 19, Xin Jie Kou Outer Street, Haidian District, Beijing 100875, P.R.China

Telephone: +86-10-58804805

E-mail: [fenglimin@bnu.edu.cn](mailto:fenglimin@bnu.edu.cn)

Table S1 Summary of camera trap surveys in annual and seasonal periods for Random Encounter Model

|  | Traps days | Wild boar | |  | Sika deer | |  | Roe deer | |
| --- | --- | --- | --- | --- | --- | --- | --- | --- | --- |
|  |  | Independent events | Group size |  | Independent events | Group size |  | Independent events | Group size |
| Annual | 83774 | 1167 | 1.81 |  | 1043 | 1.39 |  | 2714 | 1.16 |
| Summer | 41783 | 751 | 1.98 |  | 686 | 1.4 |  | 1883 | 1.16 |
| Winter | 41991 | 416 | 1.52 |  | 357 | 1.37 |  | 831 | 1.16 |
